# Assessment of toxic and endocrine potential of substances migrating from selected toys and baby products

**DOI:** 10.1007/s11356-016-7616-y

**Published:** 2016-09-23

**Authors:** Natalia Szczepańska, Jacek Namieśnik, Błażej Kudłak

**Affiliations:** Department of Analytical Chemistry, Faculty of Chemistry, Gdańsk University of Technology, 11/12 Narutowicza Str, 80-233 Gdańsk, Poland

**Keywords:** Endocrine disruptors, Toxicity, Artificial human fluids, Extraction, Toys, Migration, Children

## Abstract

Analysis of literature data shows that there is limited information about the harmful biological effects of mixture of compounds from the EDC group that are released from the surface of toys and objects intended for children and infants. One of the tools that can be used to obtain such information is appropriate bioanalytical tests. The aim of this research involved determining whether tests that use living organisms as an active element (*Vibrio fischeri*—Microtox®, *Heterocypris incongruens*—Ostrocodtoxkit F™ and the XenoScreen YES/YAS™ test of oestrogenic/androgenic activity) can be a tool for estimating the combined toxic effects induced by xenobiotics released from objects intended for children. To reproduce the conditions to which objects are exposed during their use, liquids with a composition corresponding to that of human bodily fluids (artificial sweat and saliva) were used. This research focused on the main parameters influencing the intensification of the migration process (temperature, contact time and composition of the extraction mixture). The studies aimed to estimate the endocrine potential of the extracts showed that compounds released from the surface of studied objects exhibit antagonistic androgenic activity. While on the basis of the results of Microtox® test, one can state that the largest quantity of toxic compounds are released in the first 2 h of using the object. The FTIR spectra analyses confirmed that no degradation of polymeric material took place. On the basis of the results obtained, it was unanimously concluded that contact of the object with bodily fluids may result in the release of a large number of xenobiotics, which has disadvantageous effects on the metabolic processes of the indicator organisms.

**Electronic supplementary material:**

The online version of this article (doi:10.1007/s11356-016-7616-y) contains supplementary material, which is available to authorized users.

## Introduction

The development of the chemical industry, which ensures the possibility of creating a broad range of materials that are durable, light, cheap and easy to process and produce, has undoubtedly contributed to the development of civilization and the improvement of the standard of living in society in recent years. Plastics are now the most universal and multi-functional materials used in each field of technology and every industry (Al-Natsheh et al. 2015). Despite the numerous advantages of these materials, their appearance in everyday life has had a very negative effect on the condition of the natural environment. Massive production as well as the physicochemical properties characteristic of these materials, including high durability, have contributed to a considerable increase in the amount of waste and the pollution of ecosystems by chemical compounds released from objects made from polymers (Bakir et al. 2012; Zhou et al. 2013). As proven by the results of numerous studies (aimed at finding the best possible measurement of the migration phenomenon and the factors influencing its intensification), xenobiotics are released from the surfaces of plastics not only during the degradation of these plastics but also during the use of objects made from these materials. This situation shows that everyday objects are an additional source of human exposure to various contaminants (Marć et al. 2015; Ionas et al. 2014).

Apart from polymers, auxiliary ingredients are also used for the production of polymers, which are aimed, amongst other things, at improving the physicochemical and aesthetic properties of the target products. These can include (i) fillers to increase the durability of the plastic (e.g. silica and mica; Flick 2001), (ii) plasticisers to increase the plasticity and softness of the material (e.g., soot, organic metal salts and salts or esters of phthalic acid (Bui et al. 2016, bisphenol A (BPA), its brominated derivative tetrabromobisphenol A (TBBPA) and compounds from the group of polychlorinated biphenyls (PCBs); Marć et al. 2015; Flick 2001; Zhang and Zhang 2012; Maia et al. 2009), (iii) stabilizers to prevent or delay ageing processes in the material (e.g. phenol, amine, sulphur derivatives and heavy metals; Stepek and Daoust 2012), (iv) lubricants to facilitate the processing of the polymers (paraffins and waxes), (v) agents to reduce flammability (e.g. halogenated compounds; Chen et al. 2009) and (vi) colouring agents (e.g. titanium oxide and organic pigments; Noguerol-Cal et al. 2011). Furthermore, some of them can be found in the final product as unintended contamination. The majority of synthetic compounds listed above is characterized by elevated lipophilicity; thus, they can more easily permeate biological membranes and enter live cells and, as a result, bioaccumulate in various tissues and organs (Kudłak et al. 2015a, b). This situation is made more serious by the fact that a vast majority of these compounds has properties similar to contamination from the EDC group (Endocrine Disrupting Compound; Li et al. 2010; Kudłak et al. 2015a). As a result of their oestrogen-like activity, contaminants from this group are suspected of causing numerous diseases, including fertility disorders, heart disease, cardiovascular disease and diabetes (Kudłak et al. 2015a, b). Moreover, as shown by recent reports, both BPA and phthalates may have an extra-receptor effect on organisms. The results of epigenetic studies confirmed that these compounds influence the process of histone protein methylation, causing changes in gene expression (Bernal and Jirtle 2010). The fact that these contaminants are capable of crossing the placental barrier and the blood-brain barrier, and thus, can have a negative effects on organisms in the earliest stages of life, is concerning. This supposition was confirmed in many epidemiological and experimental tests indicating a strong correlation between a mothers’ exposure to xenobiotics and the occurrence of neurodevelopmental disorders in their children, including ADHD, autism or changes in behavioural development and impairment of cognitive functions (Weiss 2012).

Hazards related to human exposure to the compounds used for the production of polymeric materials has made it necessary to develop numerous legal regulations limiting the amount of organic additives used and required the creation of guidelines concerning sample preparation methods and the methods for extraction and analysis of the analytes, e.g. directive 2009/48/EC (Directive EU 2009/48/EC) on the safety of toys and items intended for children (Ionas et al. 2016). The decreased effectiveness of the detoxification processes (related to lower activity of cytochrome P450) and the lower body weight (which influences an increase in the ratio of toxic substances per kilogramme of the child’s body) made it necessary to use higher safety requirements to take into consideration the assessment of the qualitative properties of materials intended for children (Faa et al. 2012; Mercan et al. 2015). Small-molecule ingredients of polymer materials may enter the child’s body through the respiratory, alimentary tract or transdermally. The alimentary route is undoubtedly the main source of a child’s exposure to xenoestrogens in relation to the children’s natural propensity to become familiar with the world using their mouth (Ionas et al. 2016; Guney and Zagury 2014). Atypical conditions to which polymeric materials are exposed made it necessary to modify the standard methodologies used for extracting small-molecule ingredients from them. Apart from water, special liquids are also used for extraction which allows for the reproduction of actual conditions to which objects are exposed. The most frequently used model liquids include fluids simulating the composition of bodily fluids such as: artificial saliva, artificial sweat (Özer and Güçer 2012) and artificial digestive fluids (Guney and Zagury 2014). A review available a literature data indicate that artificial saliva was used in studies aimed at quantitative determination of BPA, NP and DBP released form baby bottles and toys where the final determination was used GC-MS. The concentration of analytes were of the range of 404 ± 69 [ng/L], 154 ± 86 [ng/L] and 121 ± 33 [ng/L], respectively (Li et al. 2010; Fasano et al. 2012). Artificial saliva and sweat was used as the extraction medium to quantify the migration of heavy metals Pb and Cd from flower-shaped bracelet. Final determination were made by ICP-MS and the concentration of metals were of the range of 6100 ± 2000 [ng/g] from Pb and 139 ± 3,10 [ng/g] from Cd. (Cui et al. 2015). Supplementary Table S1 (supplementary materials) presents information about another examples of extraction methods and identification of compounds from the EDC group released from objects intended for children.

The currently adopted system for controlling the qualitative properties of objects intended for children is mostly based on results of tests obtained using instrumental methods (Ionas et al. 2016). This approach raises many concerns because hormone-imitating compounds (released from the materials tested) are present at very low concentration levels; thus, the identification and quantitative determination of all of the analytes is a large challenge for analytical chemists, and the results of instrumental analysis are often laden with errors. Additionally, a weakness of such approach is its difficulty in assessing the effects of the co-occurrence of all chemical compounds at various levels and their mutual interactions. Estimating the actual threat related to human exposure to various xenobiotics is possible only in the case of using a set of diagnostic tools with a living organism as the active element (Fuhrman et al. 2015). The possibility of estimating the cumulative burden on the samples, taking into account all relationships between individual xenobiotics, is the main reason for the increased importance of bioanalytical methods to analyse and monitor the environment. The additionally frequently significant advantage is that biological tests are characterized by relatively low cost and short time of analysis and small samples volumes. The review of specialist literature clearly indicates that no information about the use of this type of approach to assess the degree of hazards related to a child’s exposure to the contaminants released from the surface of toys is available.

In view of the aforementioned reservations, research was conducted to estimate the degree of acute and chronic toxicity and the endocrine potential of chemical substances released from objects intended for children and infants using a battery of biotests, including a test to determine the influence of the tested sample on the hormonal system. The research aimed to identify the influence of the extraction medium used, the contact time and the temperature required for releasing the endocrine compounds from the surface of selected toys and objects intended for children and infants. An attempt was made to answer the question of whether it was possible to use biotests for toxicity screening tests of commercially available products for children and infants. Research using model liquids and biological tests to assess the endocrine potential had not been used before to assess the degree of hazards on the part of products intended for children and infants.

## Methodology

### Sample collection

**Fig. 1:**
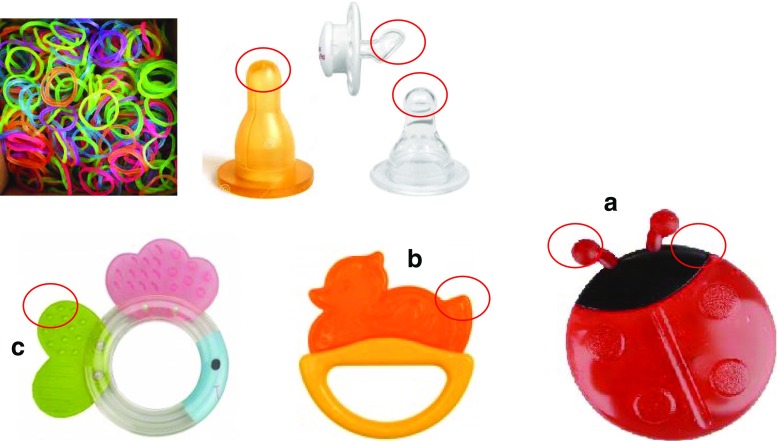
Commercially available products studied to extract easily leachable components due to contact with model extraction media

Seven basic products of everyday use intended for small children and infants were assessed for the degree of releasing endocrine compounds from their external surface. The research objects were nipples made of various materials (latex/silicone including the ones marked as “BPA free”), teethers (made of various polymeric materials, also heated to mimic the disinfection process performed by parents) and colourful rubber bands used for making decorative bracelets. All products were selected from commercially available high-quality products. In recent years, more and more fears arose on possible negative impact of colour hand bands imported from East Asia and sold in EU without valid CEs (European Conformity; The CE marking certifies that a product has met EU health, safety and environmental requirements, which ensure consumer safety). The research was not aimed at differentiating between products by various manufacturers but focused on the specification of the possible threats from these products. Some research objects were cut into pieces to fulfil another function by testing the influence of individual fragments of products on living organisms and not for a given object as a whole. These toys, and their fragments (marked with red circles, surface measured under optical microscope with microline) collected for research and marked in the manner facilitating identification of the results shown in the results’ diagrams, are presented in Fig. 1.

### Instruments, chemicals and reagents

Chemicals that were used for preparing simulant media were obtained from the following suppliers: sodium chloride (Sigma Aldrich, Germany), dipotassium phosphate (Ciech S.A., Poland), calcium chloride (Eurochem BGD, Poland), magnesium chloride, potassium chloride, potassium carbonate, lactic acid, urea (POCH S.A., Poland), ammonium hydroxide (25 % *w*/*w*), acetic acid (35–38 % *w*/*w*; Chempur, Poland) and distilled water. Chemicals used for Microtox® (2 % NaCl solution, liophilized *Vibrio fischeri*, Microtox Diulent, Microtox Acute Reagent, Osmotic Adjusting Solution, Reconstitution Solution) and Ostracodtoxkit F™ (vials with algal food for chronic toxicity tests and matrix dissolving medium, Spiruline algae, 6-well test plates, certified dormant eggs of *Heterocypris incongruens*) were purchased from ModernWater Ltd. (GB) and Microbiotest Inc. (Belgium), respectively. Reagents used for XenoScreen YES/YAS were purchased from Xenometrics G. A. (Switzerland). These were: vials containing hERα yeast cells (for YES assay) and hAR yeast cells (for YAS assay) on a filter paper, basal medium, vitamin, L-aspartic acid, L-threonine and copper sulphate solutions, CPRG (chlorophenol red-β-D-galactopyranoside**)**, vials with 17β-estradiol, 5α-dihydrotestosterone, 4-hydroxytamoxifen, flutamide and DMSO (dimethyl sulfoxide). 96-Well plates, gas-permeable plate sealers and culture flasks with gas-permeable filter cap were purchased from GenoPlastBiochemicals (Poland). All reagents were of analytical grade purity or better in the case of reagents for microbiological purposes. The instruments and equipment used during the study were: Microtox® 500 of Modern Water Ltd. (GB), microplate reader Infinite® 200 of Tecan, incubator with rotating platform, electronic multi- and single-channel pipettes (Eppendorf, Germany), analytical balance from Radwag (Poland), CP411 pH-meter from Metron (Poland) and binocular from Ceti NV (Belgium).

### Extraction

In order to release harmful ingredients from the surface of objects intended for children and infants, water and artificial sweat and saliva (to mimic in vivo realistic condition of usage as much as possible) were used. Solutions of model liquids that were used for simulation were prepared in accordance with guidelines included in standards DIN V 53160-2:2010-10 and DIN:53160-1:2010-10. Table 1 presents information about the quantitative composition of reagents necessary to prepare the extraction media.

**Table 1:** List of chemicals/composition of artificial extraction media

Simulant medium	Chemicals	Concentration [mmol/dm^3^]
Artificial sweat	Sodium chloride	86
	Lactic acid	11.1
	Urea	16.65
Artificial saliva	Sodium chloride	9.07
	Calcium chloride	1.35
	Dipotassium phosphate	4.36
	Magnesium chloride	1.79
	Potassium carbonate	3.83
	Potassium chloride	4.43
	Acetic acid	16.65

The pH values of the solutions were adjusted using a 1 % NH_3_ solution to a value of 6.8 for the artificial saliva solution and 6.5 for another extraction medium. Until the extraction process, simulation liquids were stored at +4 °C.

Due to the nature of objects studied, it was impossible to use samples with 1 dm^2^ surface for migration testing (to be in accordance with the guidance described in the standard EN1186-3:2002), for this reason smaller pieces were used for the research and results recalculated. Toys and products intended for children and infants were cut into small even pieces (to obtain a piece of ca. 3 cm^2^ total surface area from nipples and ca. 5 cm^2^ from teethers) and placed in glass vials filled with 21 cm^3^ of distilled water, artificial saliva and sweat solutions. In the case of decorative rubbers, whole object (total surface area ca. 1 cm^2^) was immersed in extractants. To accelerate the extraction process, vials were placed on a shaker table. After 30 min, 1, 2, 5 and 12 h (in the case of the extraction of decorative rubbers and nipples) and 24, 48 and 2 weeks (in the case of the extraction of teethers), 4 ml of each sample was collected. Sampling time was chosen based on the standard EN1186-1:2002. Additionally, in order to check how contact time affects the xenobiotics release rate, three supplementary sampling times were added (5, 12 and 2 weeks). To estimate the influence of the temperature on the vials containing fragments of teethers and the effects on the size of the migration stream of the toxic ingredients, the vials were heated (30 min, 100 °C—to mimic the disinfection process conducted by parents). Two sets of tests were performed. The first group of teethers was subjected to the thermal process once on the first day of extraction, while the other group was heated three times on the first, fifth and tenth day after the beginning of the extraction process. This was done to determine whether additional exposure of the tested objects to increased temperatures has a significant influence on the intensification of the migration process. The tested samples were stored at −20 °C until the biological tests were performed.

### FTIR studies

The FTIR spectra were measured for pieces of the tested material in the range of 4000–700 cm^−1^ with a *Nicolet iS50* spectrometer equipped with the Specac Quest single-reflection diamond attenuated total reflectance (ATR) accessory. Spectral analysis was controlled by the OMNIC software package.

### Procedures of bioanalytical tests

Microtox® and Ostracodtoxkit F™ have been used in order to determine the level of toxicity occurring after a short-time exposure, as well as one, which effects are observed only after a longer exposure time. Adopted battery of biotests that is utilizing organisms at different levels of the trophic chain made it possible to check whether and in what way xenobiotics affect the organisms of varying evolutionary advancement. Microorganisms represent the primary focus in the food chain; therefore, any adverse changes occurring in them, directly or indirectly, can have impact on organisms at higher trophic levels. An additional aim of the use of environmental biotests was to check whether the obtained results are consistent with those gathered using a test where genetically modified yeast with human receptors have been used as an active element.

#### Acute toxicity test Microtox®

The acute toxicity was measured with the Microtox® biotest using marine bioluminescent bacteria *Vibrio fischeri* as the active element. In this test, the assessment of the degree of toxicity is made on the basis of bioluminescent inhibition of indicator organisms. Light emission is a natural effect of metabolic process of gram-negative bacteria (Mortimer et al. 2008). At the time when factors which have a negative influence on the enzymatic activity of organisms appear, the luciferin oxidation process is inhibited, which is manifested by reduced luminescence (Parvez et al. 2006). As a result of high sensitivity of these bioluminescent organisms on the presence of toxic ingredients, they have found a broad application in environmental research to control the degree of toxicity of water, sewage, soils and sludge, medication (Czech et al. 2014), ionic liquids (Ventura et al. 2012; Hernández-Fernández et al. 2015), nanoparticles (Rossetto et al. 2014) and samples of volatile ashes (Chang et al. 2012). Figure S1 (supplementary materials) presents a diagram of the Microtox® test procedure used during the research.

#### Chronic toxicity test Ostracodtoxkit F™

The degree of chronic toxicity was determined based on the results of the Ostracodtoxkit F™ “direct contact” test using the *H. incongruens* crustacean as the indicator organism. Small organisms living in the bottom area of seas and fresh-water bodies owing to their high sensitivity to the presence of organic contaminants as well as heavy metals show high usefulness in the monitoring of the degree of contamination of water and bottom sediments (Kudłak et al. 2012). The extended duration of the test (6 days) makes it possible to assess the disadvantageous biological effects related to long-term exposure of the organism to xenobiotics and assess the exposure occurring at various developmental stages, from the larval stage to a mature specimen. The toxicity level is determined on the basis of observing two effects, including inhibition of the rate of growth and determination of the mortality of organisms as a result of contact with harmful agents (Oleszczuk 2008). Figure S2 (supplementary materials) presents a diagram of the analytical procedure used to estimate the level of chronic toxicity.

#### XenoScreen YES/YAS endocrine potential test

The XenoScreen test is used to determine hormonally active substances. This allows for the measurement of the level of oestrogenic, antioestrogenic, androgenic and antiandrogenic compounds present in the tested sample. The biotest is based on the use of genetically modified strains of *Saccharomyces cerevisiae* with built-in human oestrogenic (hERα) and androgenic receptors (hAR). Such cells are sensors of oestrogenic activity. If the substance is oestrognically active and will become bound to the oestrogenic receptor, an appropriate gene will be read in the cell, followed by a reporter gene. The reporter gene encodes β-galactosidase which participates in the process of transforming the yellow CPRG substrate (chlorophenol red-β-D-galactopyranoside) into a red product. The intensity of the red colour is directly correlated to the oestrogenic activity of the substance. If this test is used, it is possible to obtain information about the oestrogenic activity and not the quantity or concentration of substances indicated by the endocrine properties (Kudłak et al. 2015a, b; Thomas et al. 2009).

The test was performed on the basis of instructions delivered by the manufacturer, however, with certain modifications. Figure S3 (supplementary materials) presents the diagram of the procedure used for determinations.

Positive and solvent control were included in which experiment. Final hormone concentration in positive controls ranged from 0.10 to 100 μM. For the oestrogenic activity, the growth factor (G) and induction ratio (I_R_) according to the equations:

1 $$ G=\frac{A_{690,S}}{A_{690,N}} $$

2 $$ {I}_R=\frac{1}{G}\bullet \frac{\left({A}_{570,S}-{A}_{690,S}\right)}{\left({A}_{570,N}-{A}_{690,S}\right)} $$

where A_690, S_ and A_570, S_ is absorbance of samples, respectively, at 690 and 570 nm and A_690, N_ and A_570, N_ is absorbance of the solvent control, respectively, at 690 and 570 nm. The determinations were repeated three times.

For the data assessment, the criterion was adopted that the tested sample has agonistic YES/YAS properties if the value of the induction coefficient ≥1.5 (for control solutions) and shows antagonistic YES/YAS properties if the value of the induction factor ≤66.7 % of the value obtained for the control sample.

### Quality assurance/quality control

For quality assurance of proper test run, the following parameters according to producer guidelines were assumed: for Microtox®, I_0_ of bacterial suspension >70 U (chromium sulphate was used as a positive control of bacterial stock suspension test run); for control organisms in Ostracodtoxkit F™, mean growth increment >400 μm while their mortality <20 % and for Xenoscreen YES/YAS, the OD_690_ of yeasts culture should be >0.3. In all the cases, these requirements were fulfilled.

## Results and discussion

**Fig. 2:**
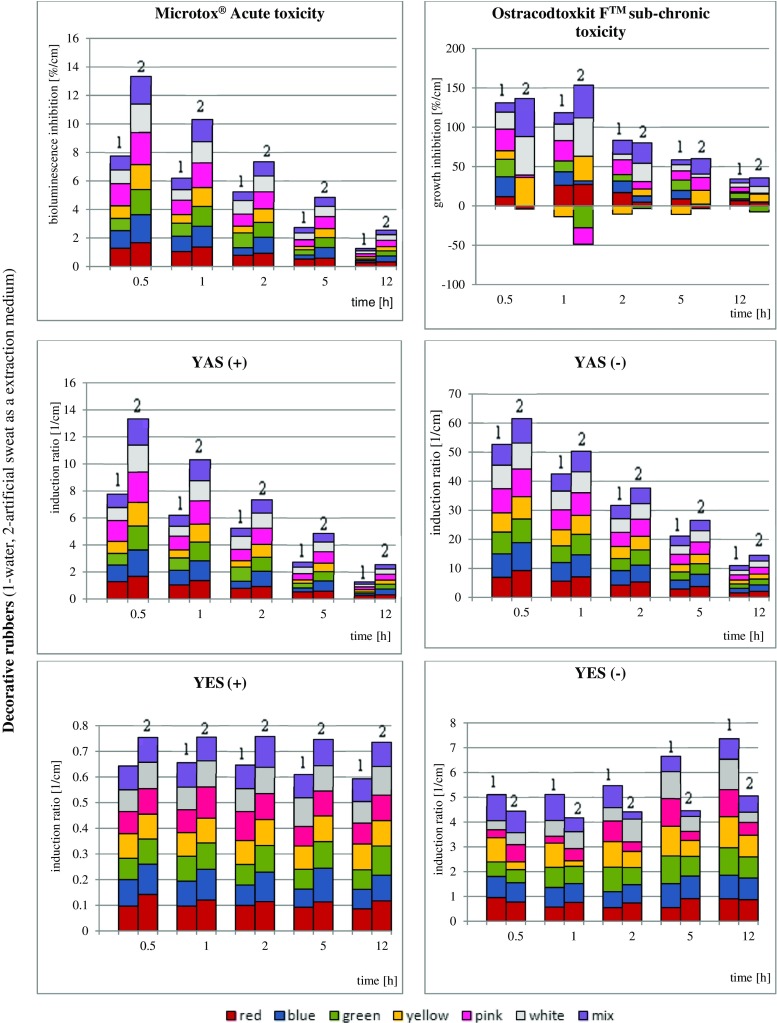

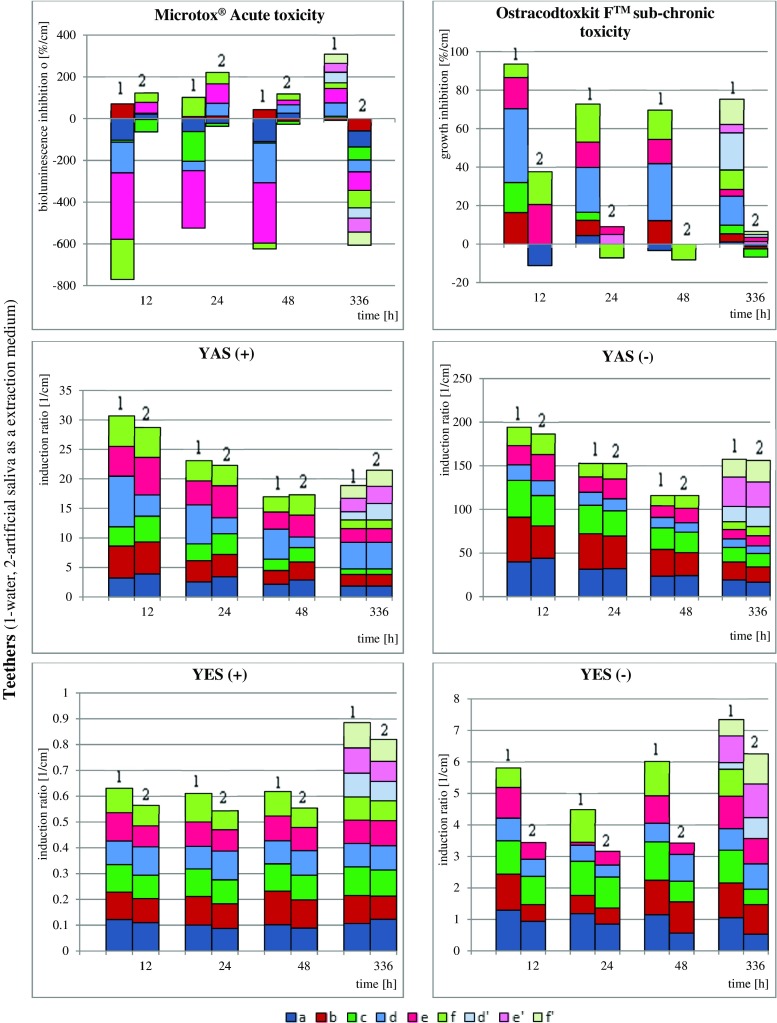

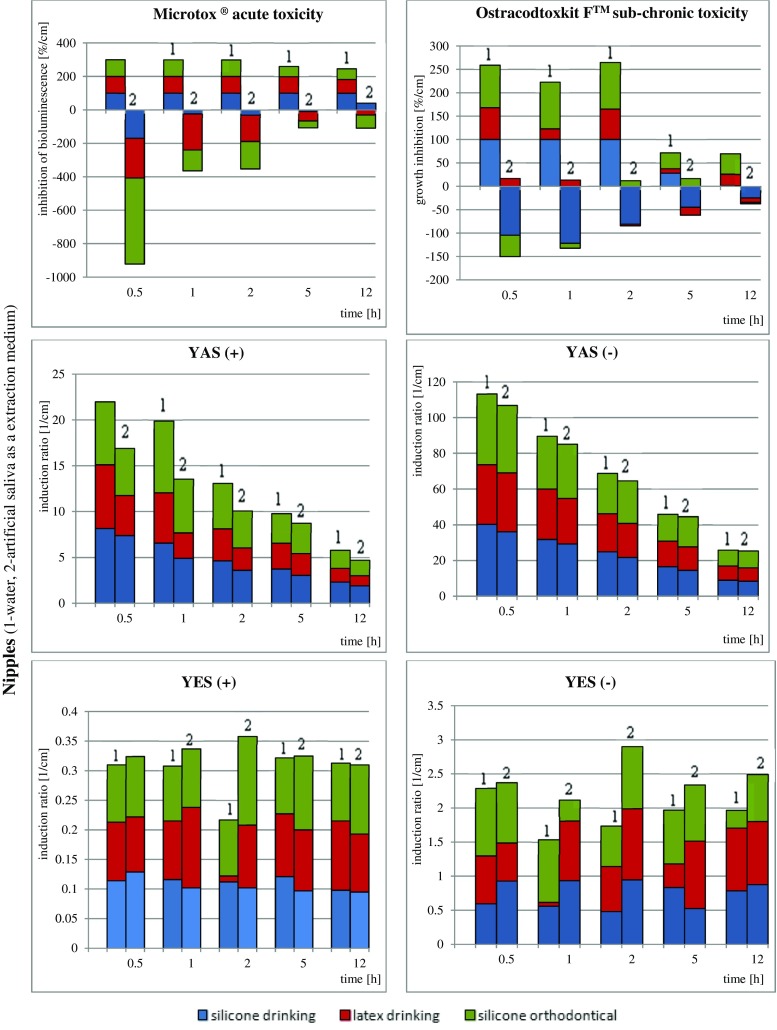
**a** Results of biological assays of decorative rubbers extracts samples. **b** Results of biological assays of teether extract samples. **c** Results of biological assays of nipples extracts samples

The results of biological studies are graphically presented in Fig. 2; FTIR spectra of all of the samples are presented in supplementary Fig. S4.

The results are calculated and presented in relation to specific surfaces of the product that underwent extraction with a specific volume of the given extraction medium $\frac{\mathrm{effect}\bullet \mathrm{sample}\ \mathrm{surface}}{\mathrm{extractant}\ \mathrm{volume}}\left[\frac{\%}{\mathrm{cm}}\mathrm{or}\ \frac{1}{\mathrm{cm}}\right]$. As the static experimental design has been prepared, such recalculation has been performed to unify the results because the migration depends on varying (decreasing with time) extraction medium volume and constant sample surface (calculated as entire surface of sample being in contact with the extraction media). In this way, the dilution of xenobiotics with time and reduced stimulant volume was avoided. Because studying all commercially available pacifiers, teethers and nipples is not possible due to vast number of producers, only high-quality products (with European Commission safety certificates and labelled as bisphenol A free) were purchased to perform experiments. Decorative bands were selected as additional research object due to recent increase of scientific reports (in 2014 and 2015) on negative impact of low-quality bands on skin irritation incidences in Europe. Certainly there is no direct correlation between results of bioassays and human health exposure, however, the final outcome will depend on the extent to which the humans will be exposed.

The nature of materials used to prepare the toys and pacifiers was confirmed by the ATR-FT-IR measurements. The data collected in Table 2 resulted from the comparison of each measured spectrum with the commercially available libraries (Thermoscientific). The identification of polymers listed in the Table 2 was conducted on the basis of the match factor between their FT-IR patterns and the measured spectra as well as the careful assessment of their spectral features i.e. the presence of characteristic bands. Results and comments on FTIR spectra are given in Table 2. FTIR spectral studies were conducted prior to and after the extraction. No degradation of material was stated proving that only leachable ingredients were responsible for observed toxicological response.

**Table 2:** The ATR-IR results of characterization of the samples studies

Object	Material	Match factor
Yellow decorative rubber	Styrene butadiene block polymer	79.96^a^
Blue decorative rubber	Styrene butadiene block polymer	76.50^a^
Pink decorative rubber	Styrene butadiene block polymer	81.13^a^
Red decorative rubber	Styrene butadiene block polymer	75.58^a^
White decorative rubber	Styrene butadiene block polymer	80.12^a^
Green decorative rubber	Styrene butadiene block polymer	82.07^a^
Teether (object (b) in Fig. 1.)	Poly(thylene:propylene:diene)	85.47^b^
	Poly(ethylene:propylene)	84.26^b^
	Polypropylene	82.50^a^
Teether (object (c) in Fig. 1)	Poly(thylene:propylene:diene)	94.78^b^
Teether (object (a) in Fig. 1)	Poly(vinyl acetate:ethylene) - probably different ratio of vinylacetate and ethylene in the model spectrum	35.89^b^
	Ethylene vinyl acetate polymer	78.33^a^
Teether (object (a) in Fig. 1., after extraction with artificial saliva)	Polystyrene atactic	90.52^b^
	Poly(styrene acrylonitrile:methyl methacrylate)	60.76^b^
Silicone drinking nipple	Poly(dimethylsiloxane) or other polysiloxane material.	33.69^a^
Latex drinking nipple	Poly(ethylene:propylene)—admixture of aromatic hydrocarbons (probably polystyrene)	77.95^b^
Silicone pacifier	Poly(dimethylsiloxane) or other polysiloxane material.	31.66^a^

Library: a HR Nicolet Sampler Library (Thermoscientific), b Hummel Polymer Sample Library (Thermoscientific)

As presented in Fig. 2., in the case of the acute toxicity determination of decorative rubber bands extracts, there is a noticeable decrement of bioluminescence inhibition with extraction time.

It can be concluded that acute risk posed by extractable xenobiotics is the highest at first 2 h of contact between object and person using it. Similar correlation can be observed in case of results of sub-chronic toxicity determination with Ostracoda (although some 1-h-extracted samples (on green and pink rubbers) exhibit positive impact on bioassaying organisms) and Xenoscreen YES/YAS (agonistic androgenic behaviour of all objectives was observed, both for water and artificial sweat extraction media). Further studies must be conducted in case of white-coloured bands to explain whether their elevated toxicity results from zinc oxide presence as pigment admixture. One can also notice the positive impact of extractable ingredients in samples extracted with artificial sweat, with the exception in the case of the blue-coloured band, which was toxic even after a short contact time.

Interesting results were obtained in the studies on andro- and oestrogenic activities of teether extracts (see Fig. 2b). There is a clear impact of extraction medium on the efficiency of degradation of the given objects of interest, as well as of the temperature and extraction time. As the methodology for the extraction of the teethers was adjusted for possible heating with boiling water (100 °C, to mimic the disinfection of these objects by parents), it can be noticed that these doubly heated teether extracts show elevated response of bioassays used (d’, e’, f’ objects in Fig. 2b), proving that the degradation and release of endocrine-potent molecules from object studied occurs—which may have negative impact on endocrine system of pupils (increasing antagonistic activity of androgen receptors). High toxicity can be observed for a cooling teether extracts, where the sub-chronic toxicity levels after water extraction exceeds 75 %.

The biggest attention should be however paid to results of drinking nipples toxicological studies (ref. to Fig 2c), where in case of aqueous extracts of drinking and orthodontic nipples, a very high level of toxicity (100 %) was observed. Such an elevated inhibition of bacterial bioluminescence and growth of Ostracods proves that this stimulant medium causes a release of a significant amount of small-particle ingredients of latex and silicone material. The extension of the extraction time (in the case of artificial saliva as the extraction medium) results in increasing toxicity values. Similar observations were made for the determination of toxicity extracts from drinking and orthodontic nipples with *H. incongruens*. These observations prove that these objects cause significant inhibition of enzymatic activity of test organisms and are a source of pollution to ecosystems and harm to human beings. Interestingly, the aqueous extracts in the case of drinking nipples and teethers exhibit higher toxicity levels when compared to samples extracted with artificial saliva.

## Conclusions

The main objective of the research project which results are presented here was to assess the cumulative toxic effects of a mixture of xenobiotics released from the toys and objects intended for children (nipples, pacifiers, teethers and colour decorative bands) using a battery of biotests (Microtox®, Ostracodtoxkit F™ and Xenoscreen YES/YAS). Artificial sweat and saliva were used as the simulation media while aqueous extracts were considered as reference medium. The analyses of FTIR spectra of samples (prior to and after extraction process) confirmed that no degradation of polymers took place during the extraction processes, and thus, only loosely bound and easily extractable ingredients migrating from polymeric materials were responsible for toxicological results observed.

The results obtained justify the statement that as a result of contact between the tested object and the simulation liquids, the small-molecule ingredients of samples used at the production stage of items intended for children were rinsed out. For the majority of samples (where distilled water was used as the extraction medium), regardless of the extraction time, a reduction in the bioluminescence level was noticed for the Microtox® toxicological studies. The results of tests based on the β-galactosidase secretion and its activity (Xenoscreen YES/YAS) after contact with samples studied confirm antagonistic androgenic activity of sample ingredients what is of particular importance in case of items devoted for children. On the basis of the data obtained it can be concluded that the disadvantageous biological effect induced by xenobiotics released from the objects studies (except nipples extracts results with Microtox® studies) is decreasing with the extension of the extraction time. This situation may indicate the fact that as a result of longer contact time with the extraction medium, the released compounds may be transformed into derivatives characterized by a lower degree of biological activity.

The data obtained constitute an important source of information not only about the exposure of children to compounds belonging to the EDC group, but also in the field of environmental studies. The observed unfavourable biological effects induced by the released compounds show that it is necessary to continue research on the estimation of the degree of toxicity of xenobiotics used for the production of polymeric materials and the identification of the actions aimed at including bioanalytical methodologies at the stage of controlling qualitative properties of products intended for children and infants.

## Electronic supplementary material

ESM 1 (PDF 1.15 mb)
