# Supplementary material for: Assessment of toxic and endocrine potential of substances migrating from selected toys and baby products
Source: Environ Sci Pollut Res Int. 2016 Sep 23;23(24):24890–900. doi: 10.1007/s11356-016-7616-y (PMC5124054; doi:10.1007/s11356-016-7616-y)
Supplement: Supplementary file 1 — (PDF 1.15 mb) [file 11356_2016_7616_MOESM1_ESM.pdf]

Table S.1. Information on extraction methods and identification of EDC compounds in products devoted for children

Sample type	Analytes	Sample preparation	Final determina-tion	Concentration determined	Ref
toys	DEHP	LLE – artificial saliva	GC-MS	1,29 ± 4,78 [ng/g]	Özer, Gücer, 2011
toys	benzene	μ-CTE™	TD-GC-MS	0,45 ± 0,33 [ng/g]	Marć *et al.*, 2015
	tolune			3.3 ± 2.6 [ng/g]	
children food packagings	DBP	QuEChERS	GC-MS	1.59 ± 0,16 [ng/g]	Fasano *et al.*, 2012
	NP			9.61 ± 1.93 [ng/L]	
	BPA			0,87 ± 0,15 [ng/g]	
baby's bottle	DBP	SPE, LLE artificial saliva	GC-MS	121 ± 33 [ng/L]	Li *et al.*, 2010
	NP			154 ± 86 [ng/L]	
	BPA			404 ± 69 [ng/L]	
toys	Pb	LLE – artificial saliva	ICP-OES	5000 ± 700 [mg/kg]	Godoi *et al.*, 2011
	Cd			0,42 ± 0,57 [mg/kg]	
flower-shaped bracelet	Pb	LLE –acetic acid solution	ICP-MS	6100 ± 2000 [ng/g]	Ciu *et al.*, 2015
	Cd			139 ± 3,10 [ng/g]	
toys	DBDPE	Soxhlet extraction (acetone/hexane)	GC-MS	34300 [ng/g]	Chen *et al.*, 2009
baby bottle	BPA	LLE – water with dishwashing detergents	GC-MS	987 [ng/L]	Maia *et al.*, 2009
baby bottle	BPA	LLE - distilled water	CL-ELISA	4,5 [μg/mL]	Maiolini *et al.*, 2014
toys	DEHP	LLE artifiial sweat	GC-MS	94,14 [μg/g]	Weiss, 2012

BPA – Bisphenol A, CL-ELISA - chemiluminescence enzyme-linked immunosorbent assay, DBP – dibuthyl phthalate, DEHP – Bis(2-ethylhexyl) phthalate, GC-MS- gas chromatography mass spectrometry, ICP-OES- inductively coupled plasma optical emission spectrometry, LLE - liquid-liquid extraction, NP - nonylphenol DBDPE – decabromodiphenyl ethane, TD – thermal desorption, μ-CTE – Micro-Chamber/Thermal Extraction

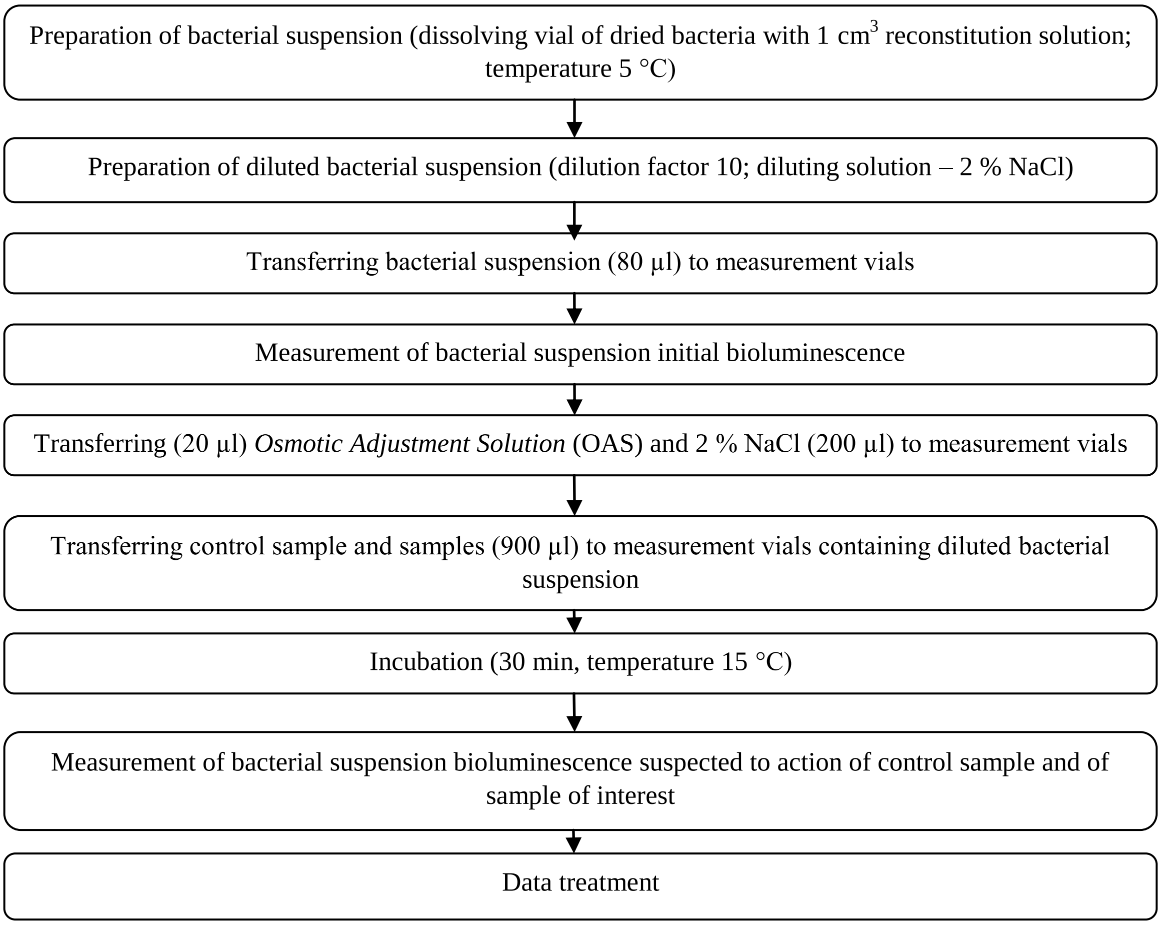

Fig. S1. Analytical procedure of acute toxicity determination.

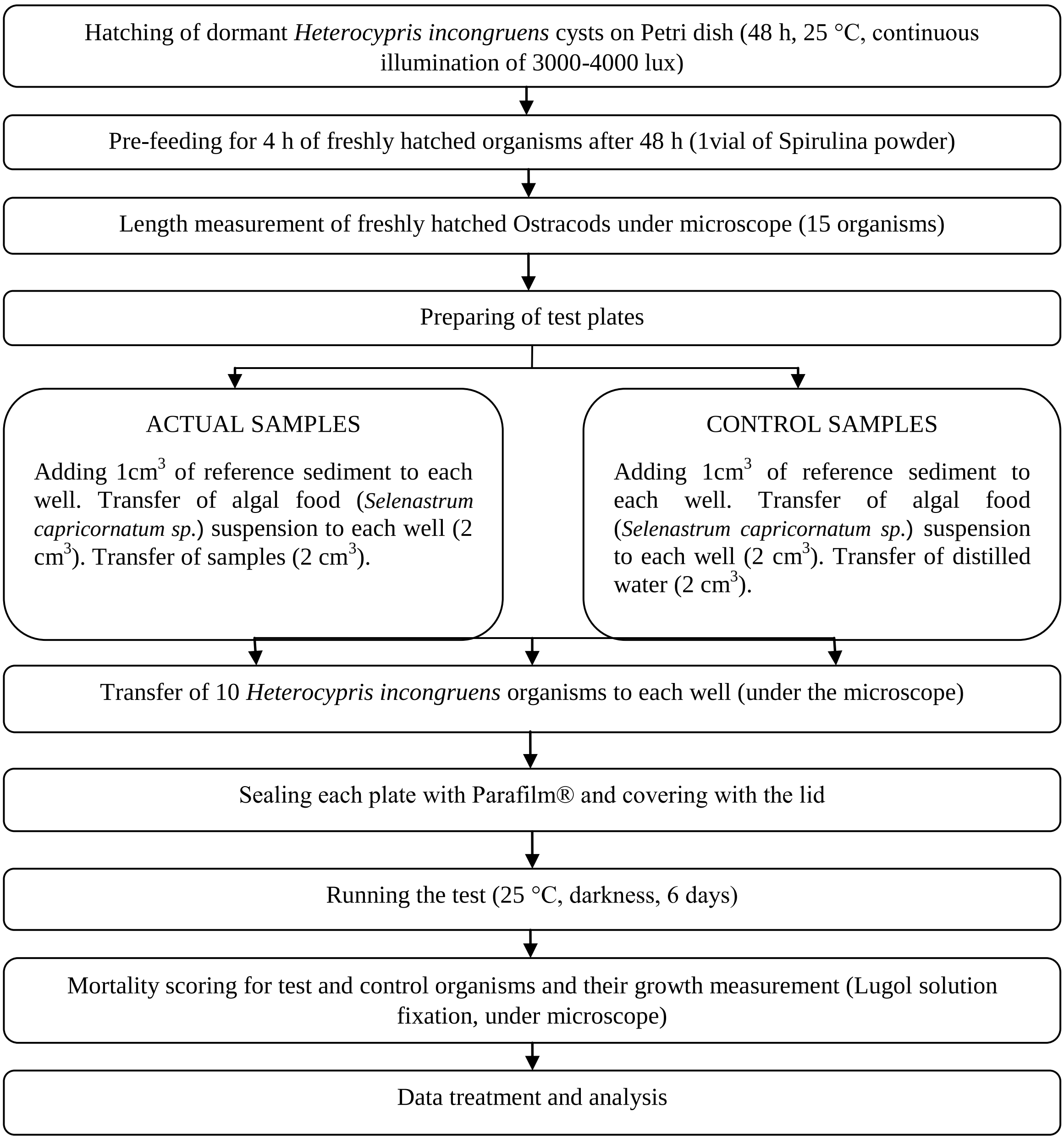

Fig. S2. Schematic presentation of *Heterocypris incongruens* sub-chronic toxicity determination procedure.

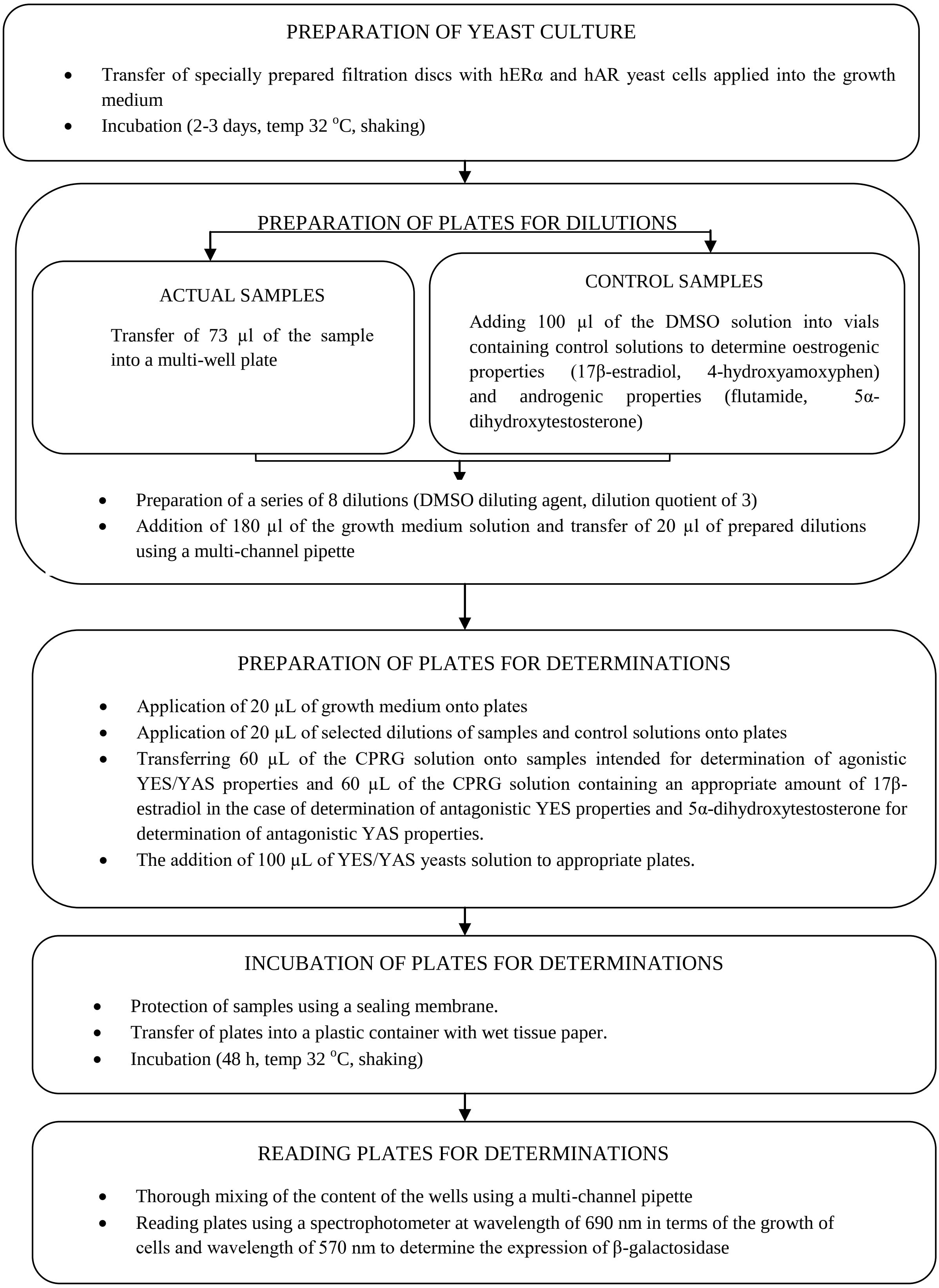

Fig. S3. Diagram of the procedure of the oestrogenic and androgenic activity

Fig.S.4. IR spectra of materials used to produce toys and baby products.

a)

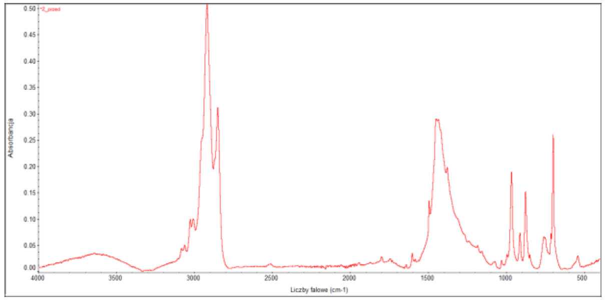

b)

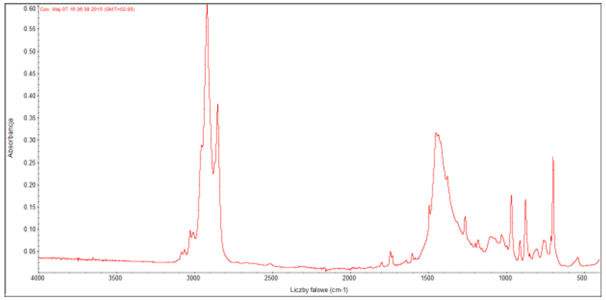

c)

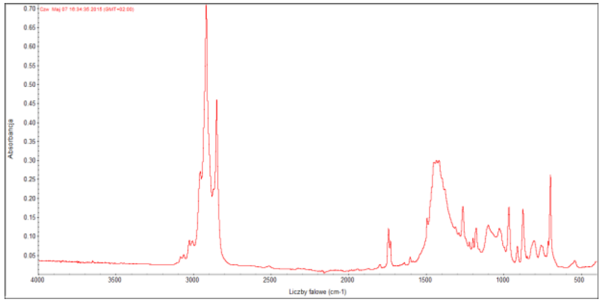

S.4.1. Yellow rubber: a) before extraction, b) after extraction with water, c) after extraction with artificial sweat solution.

a)

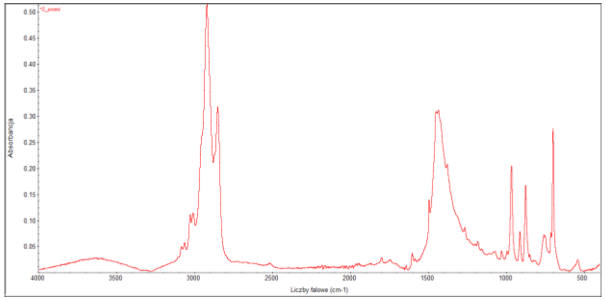

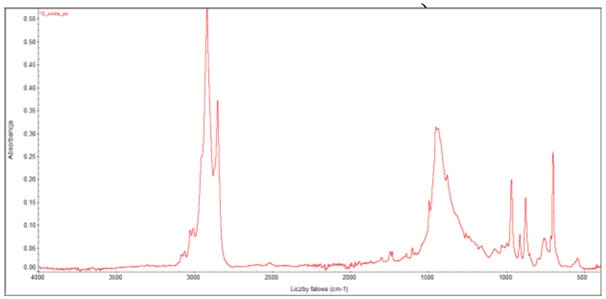

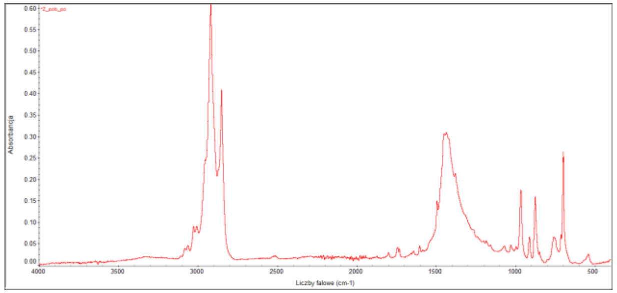

S.4.2. Green rubber: a) before extraction, b) after extraction with water, c) after extraction with artificial sweat solution.

a)

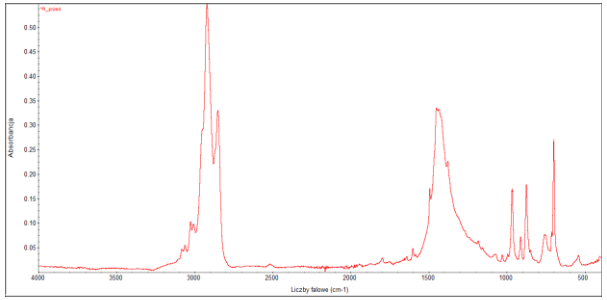

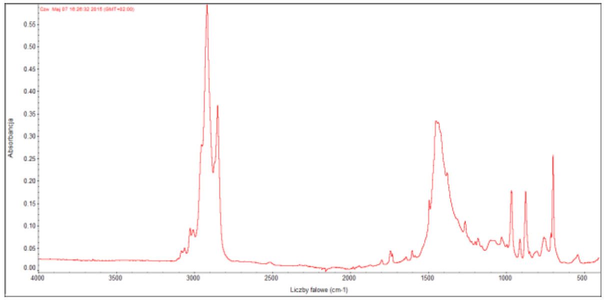

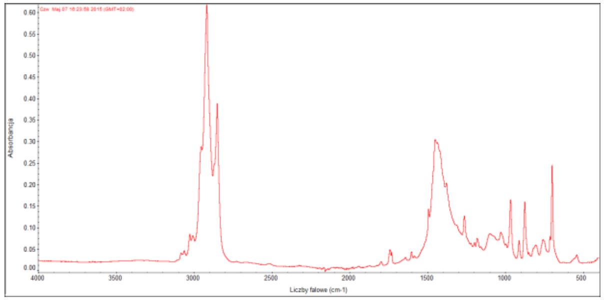

S.4.3. Pink rubber: a) before extraction, b) after extraction with water, c) after extraction with artificial sweat solution.

a)

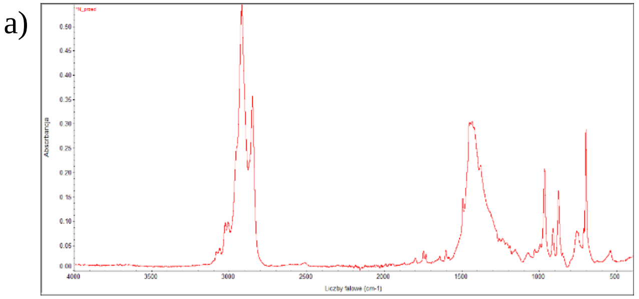

b)

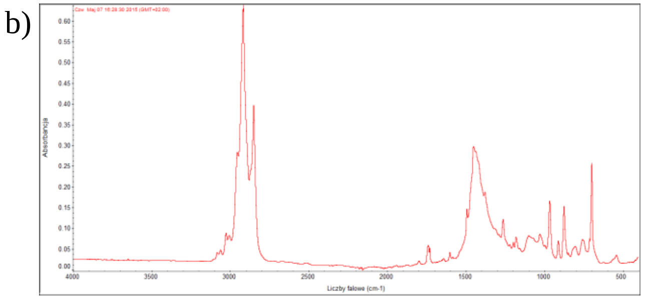

c)

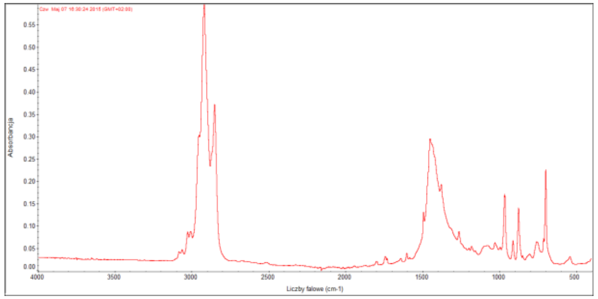

S.4.4. Blue rubber: a) before extraction, b) after extraction with water, c) after extraction with artificial sweat solution.

a)

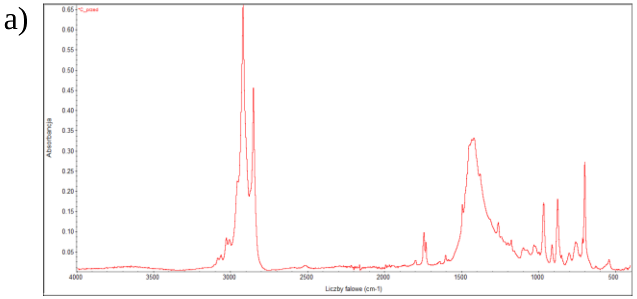

b)

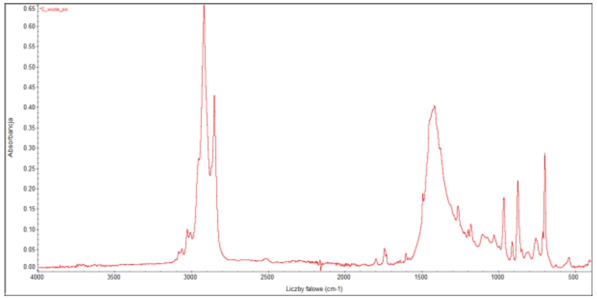

c)

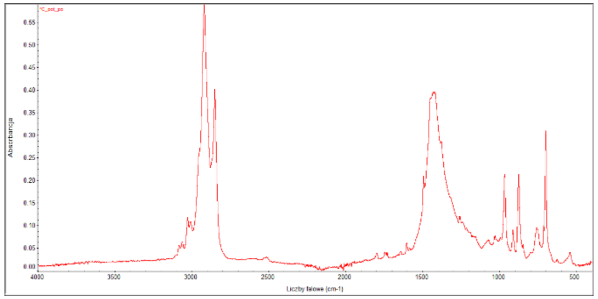

S.4.5. Red rubber: a) before extraction, b) after extraction with water, c) after extraction with artificial sweat solution.

a)

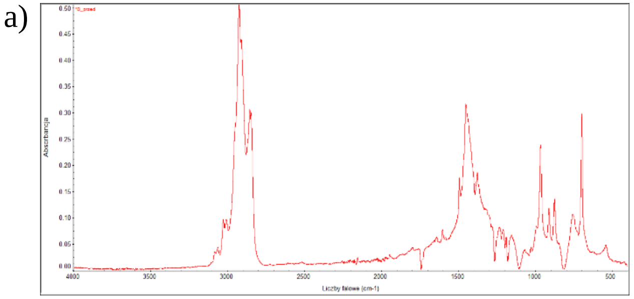

b)

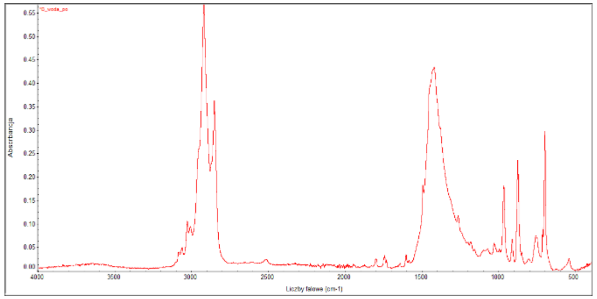

c)

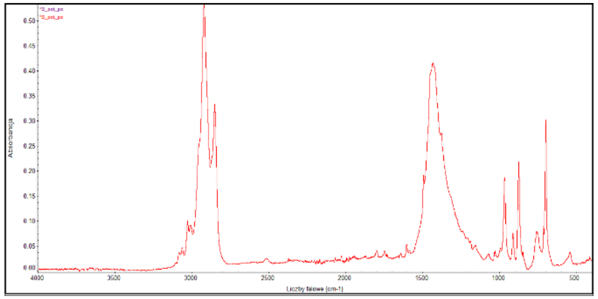

S.4.6. White rubber: a) before extraction, b) after extraction with water, c) after extraction with artificial sweat solution.

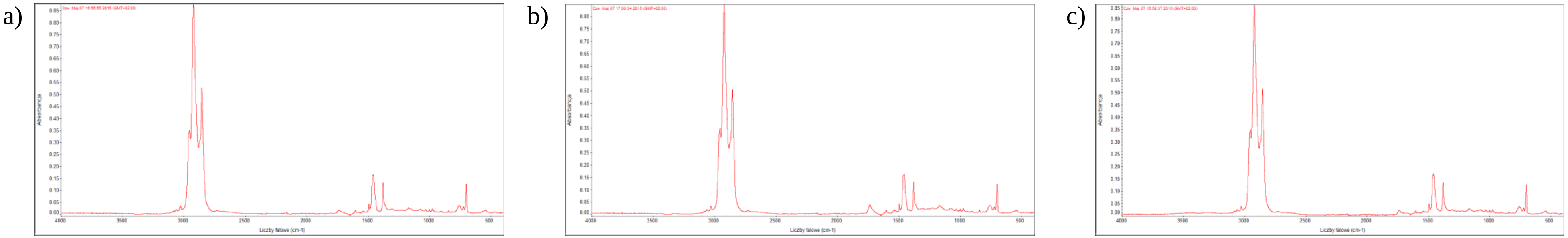

S.4.7. Rattle with elastic teether: a) before extraction, b) after extraction with water, c) after extraction with artificial saliva solution.

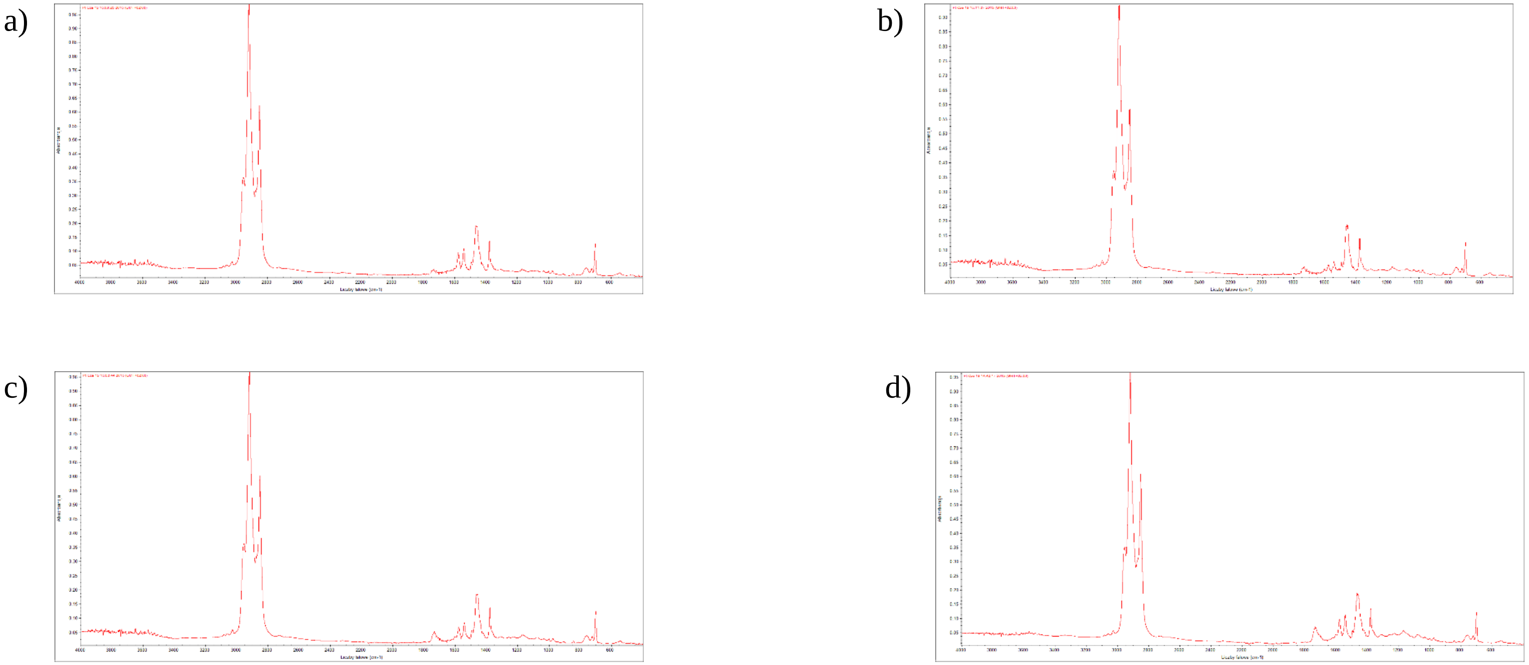

S.4.8. Rattle with elastic teether: a) after extraction with artificial saliva solution, heated to 100 ^{o}C, b) extraction with artificial saliva solution, doubly heated to 100 ^{o}C, c) after extraction with water, heated to 100 ^{o}C c) extraction with water, doubly heated to 100 ^{o}C

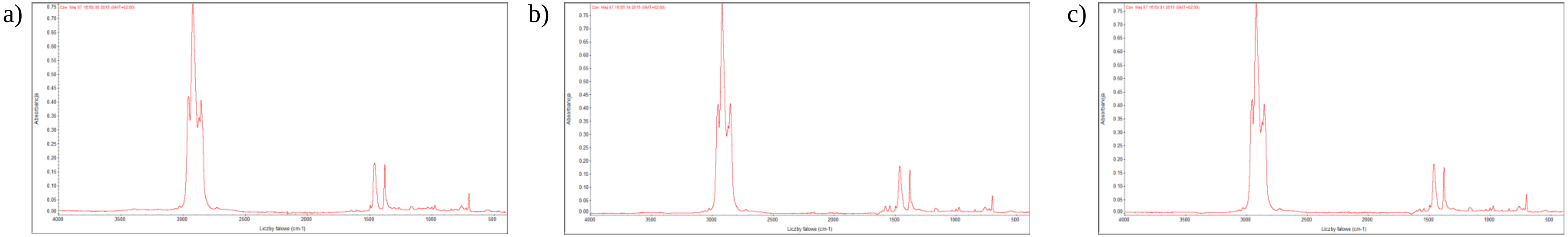

S.4.9. Rattle with soft bite teether: a) before extraction, b) after extraction with water, c) after extraction with artificial saliva solution.

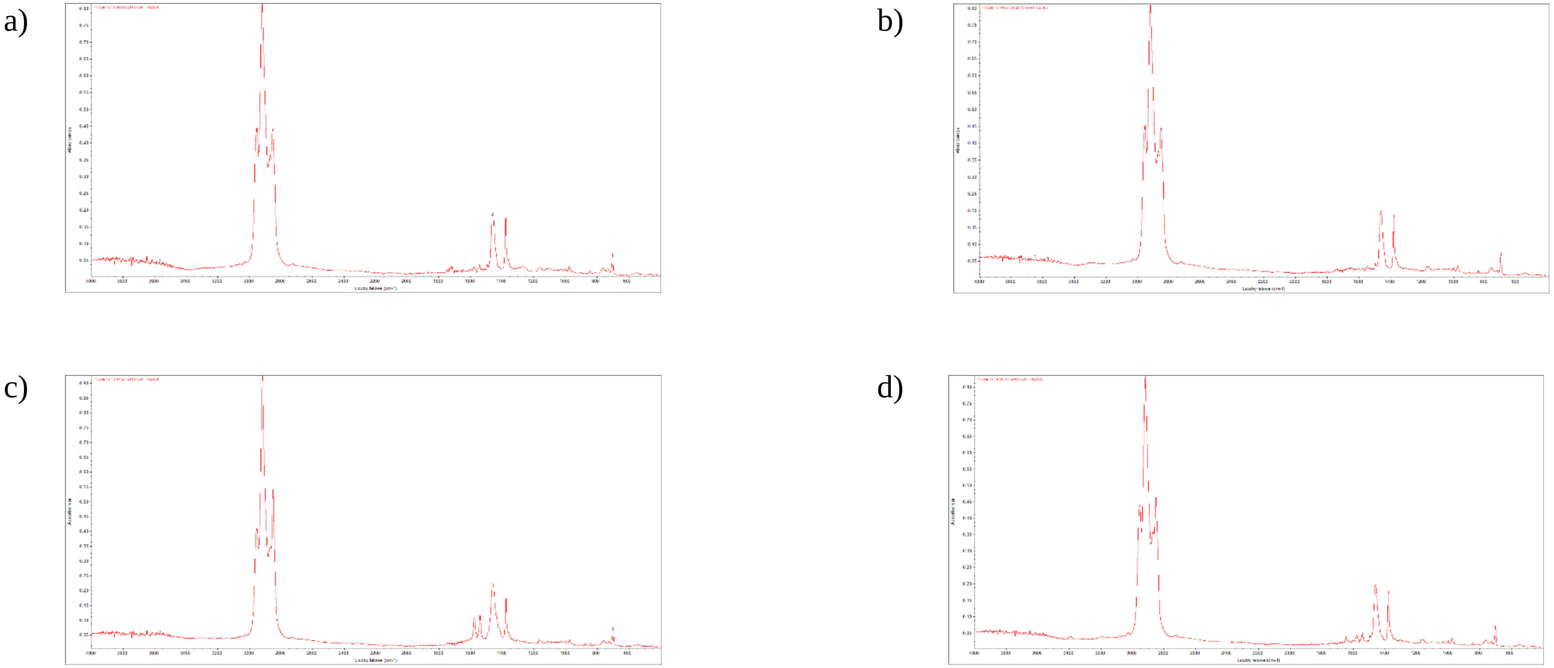

S.4.10. Rattle with soft bite teether: a) after extraction with artificial saliva solution, heated to 100 ^{o}C, b) extraction with artificial saliva solution, doubly heated to 100 ^{o}C, c) after extraction with water, heated to100 ^{o}C. c) extraction with water, doubly heated to 100 ^{o}C

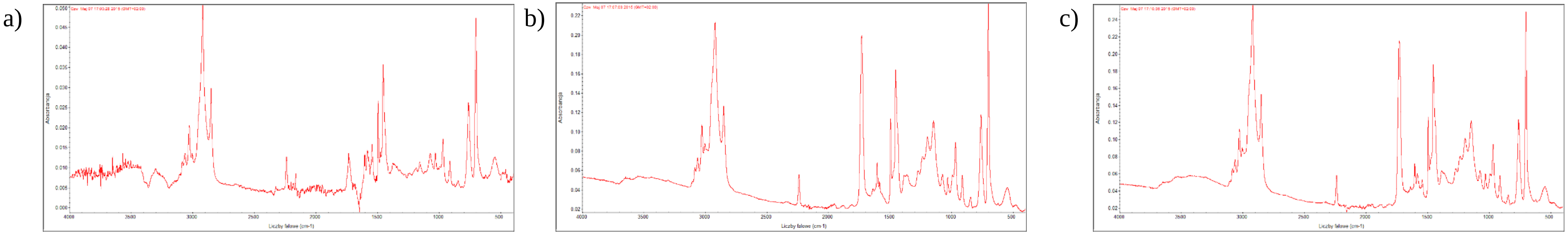

S.4.11.Cooling teether, hard part: a) before extraction, b) after extraction with water, c) after extraction with artificial saliva solution.

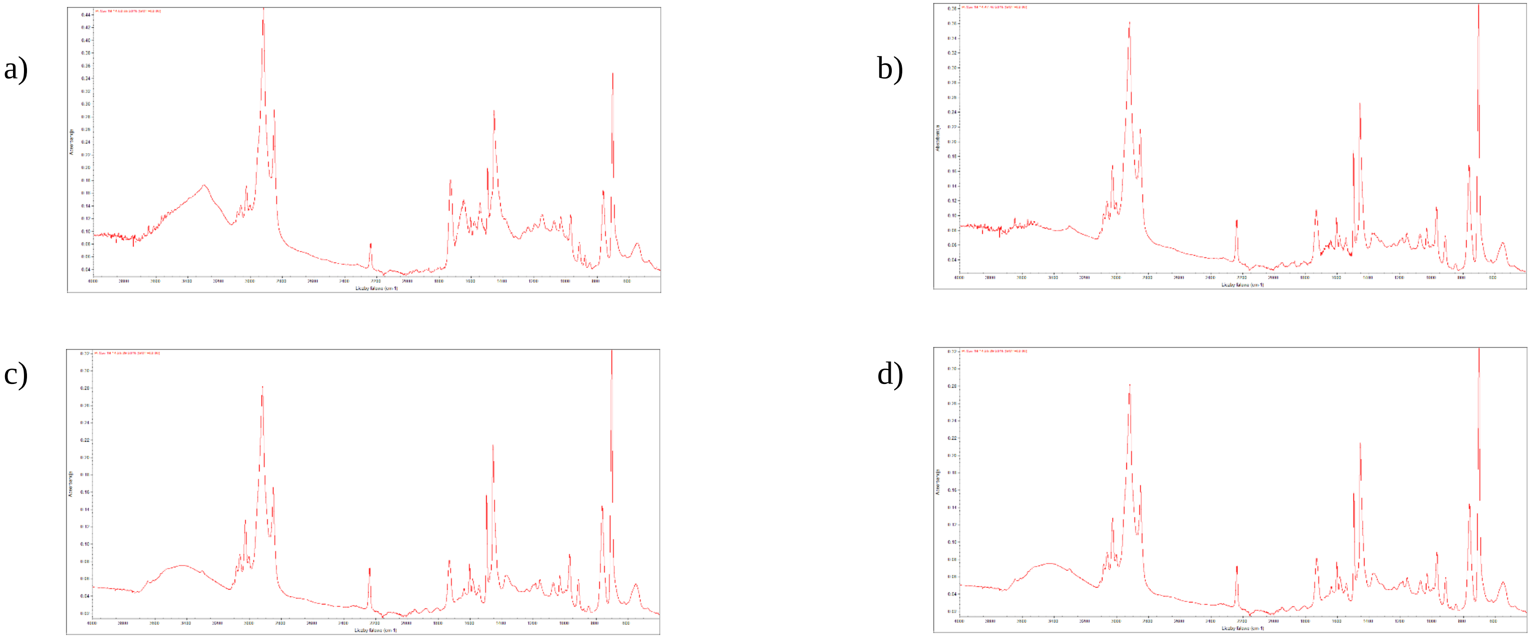

S.4.12.Cooling teether, hard part: a) after extraction with artificial saliva solution., heated to 100 ^{o}C, b) extraction with artificial saliva solution, doubly heated to 100 ^{o}C, c) after extraction with water, heated to 100 ^{o}C c) extraction with water, doubly heated to 100 ^{o}C

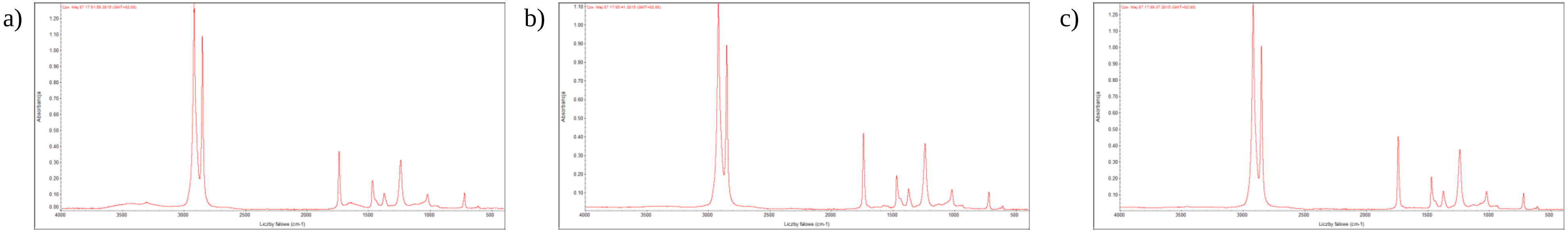

S.4.13. Cooling teether: a) before extraction, b) after extraction with water, c) after extraction with artificial saliva solution.

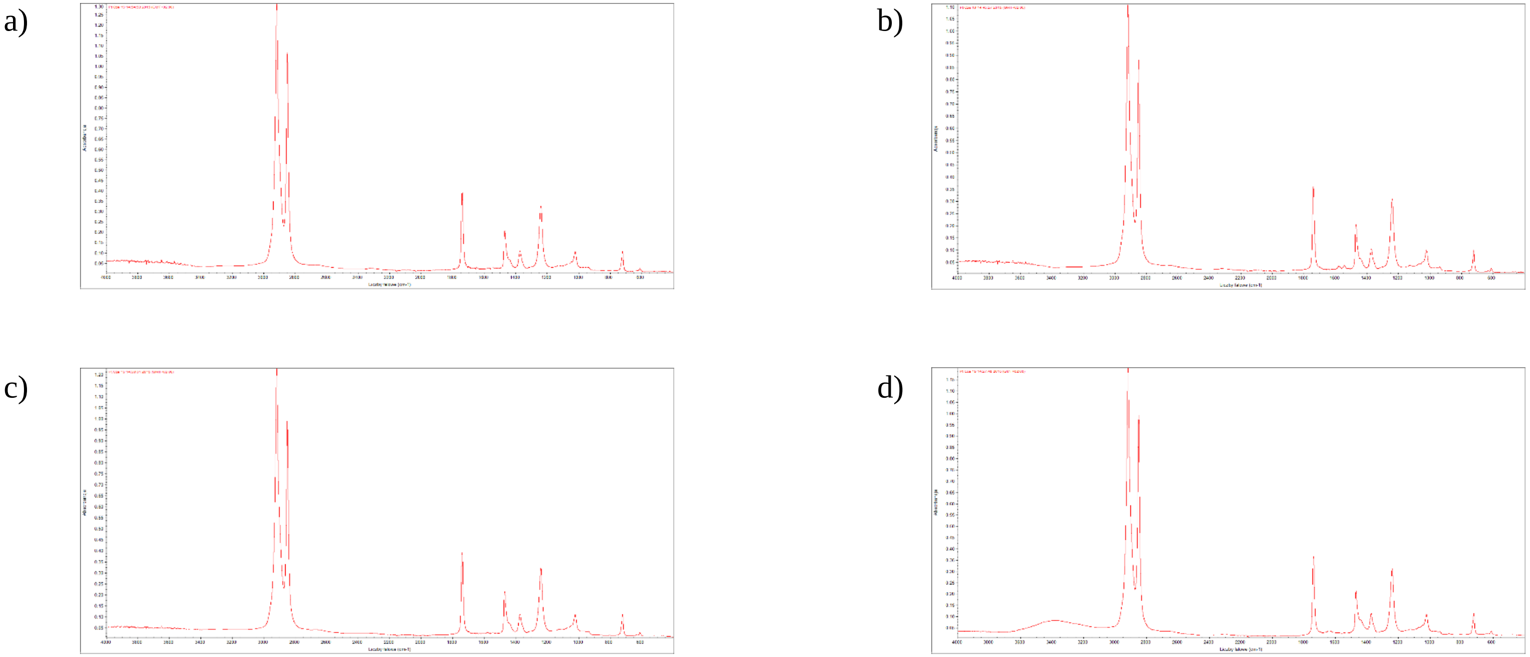

S.4.14. Cooling teether: a) after extraction with artificial saliva solution, heated to 100 ^{o}C, b) extraction with artificial saliva solution, doubly heated to 100 ^{o}C, c) after extraction with water, heated to 100 ^{o}C, c) extraction with water, doubly heated to 100 ^{o}C

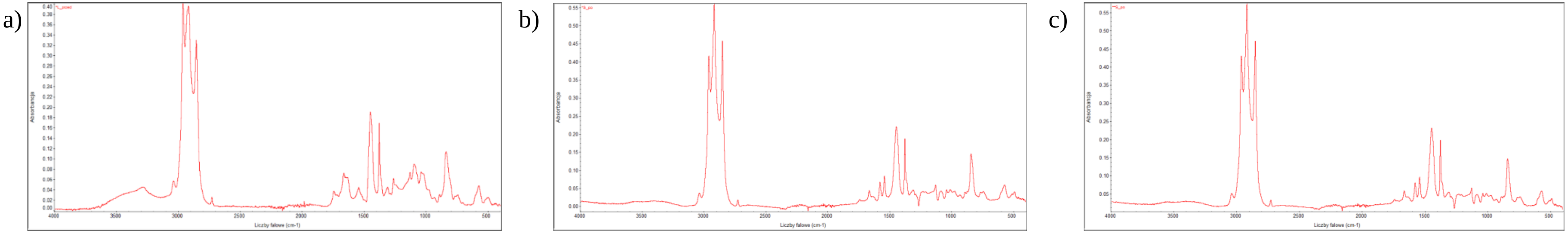

S.4.15. Latex nipple: a) before extraction, b) after extraction with water, c) after extraction with artificial saliva solution.

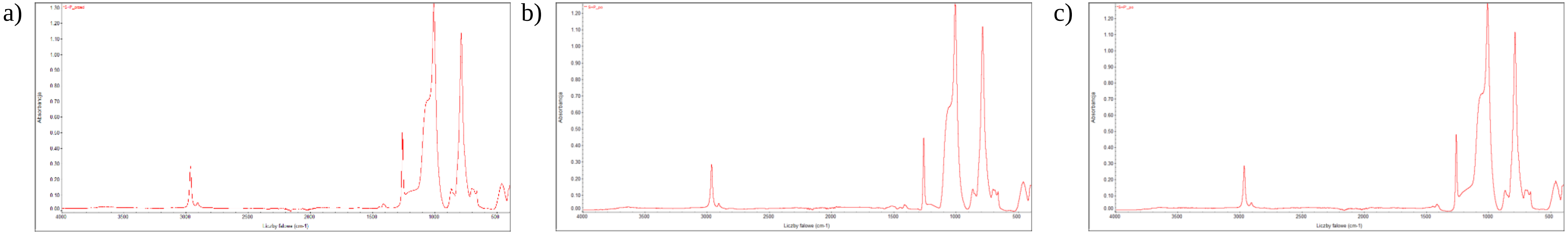

S.4.16. Silicone nipple: a) before extraction, b) after extraction with water, c) after extraction with artificial saliva solution.

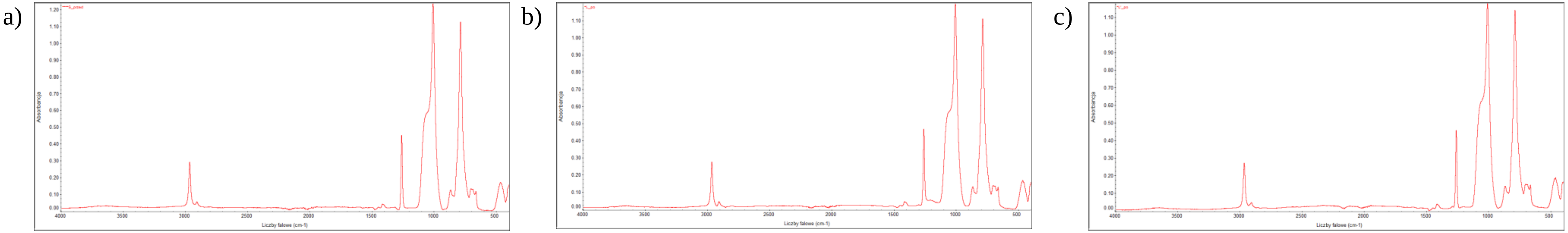

S.4.17. Silicone drinking nipple: a) before extraction, b) after extraction with water, c) after extraction with artificial saliva solution
